# A new small duckbilled dinosaur (Hadrosauridae: Lambeosaurinae) from Morocco and dinosaur diversity in the late Maastrichtian of North Africa

**DOI:** 10.1038/s41598-024-53447-9

**Published:** 2024-02-13

**Authors:** Nicholas R. Longrich, Xabier Pereda-Suberbiola, Nathalie Bardet, Nour-Eddine Jalil

**Affiliations:** 1 Department of Biology and Biochemistry and Milner Centre for Evolution, University of Bath, Bath, BA2 7AY UK; 2 Departamento de Geología, Facultad de Ciencia y Tecnología, Universidad del País Vasco/Euskal Herriko Unibertsitatea, Apartado 644, 48080 Bilbao, Spain; 3 CR2P, Centre de Recherche en Paléontologie-Paris, CNRS-MNHN-Sorbonne Université, Muséum National d’Histoire Naturelle, CP38, 57 rue Cuvier, 75005 Paris, France; 4 Museum of Marrakech (Museum of Natural History of Marrakesh, Univ. Cadi Ayyad), Marrakesh, Morocco

**Keywords:** Palaeontology, Phylogenetics

## Abstract

In the Late Cretaceous, northern and southern hemispheres evolved distinct dinosaurian faunas. Titanosaurians and abelisaurids dominated the Gondwanan continents; hadrosaurids, ceratopsians and tyrannosaurs dominated North America and Asia. Recently, a lambeosaurine hadrosaurid, *Ajnabia odysseus*, was reported from the late Maastrichtian phosphates of the Oulad Abdoun Basin Morocco, suggesting dispersal between Laurasia and Gondwana. Here we report new fossils from the phosphates of Morocco showing lambeosaurines achieved high diversity in the late Maastrichtian of North Africa. A skull represents a new dwarf lambeosaurine, *Minqaria bata*. *Minqaria* resembles *Ajnabia odysseus* in size, but differs in the ventrally positioned jugal facet and sinusoidal toothrow. The animal is small, ~ 3.5 m long, but the fused braincase shows it was mature. A humerus and a femur belong to larger hadrosaurids, ~ 6 m long, implying at least three species coexisted. The diversity of hadrosaurids in Europe and Africa suggests a dispersal-driven radiation, with lambeosaurines diversifying to take advantage of low ornithischian diversity. African lambeosaurines are small compared to North American and Asia hadrosaurids however, perhaps due to competition with titanosaurians. Hadrosaurids are unknown from eastern Africa, suggesting Moroccan hadrosaurids may be part of a distinct insular fauna, and represent an island radiation.

## Introduction

**Figure 1:**
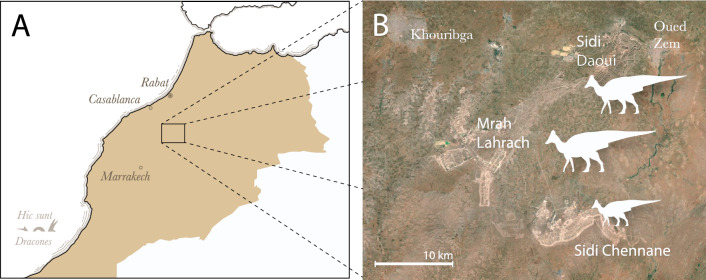
Map showing localities that produced the fossil specimens described in this paper. (**A**), location of the Oulad Abdoun Basin, central Morocco; (**B**), localities of hadrosaurids recovered from Sidi Daoui, Mrah Lahrach, and Sidi Chennane.

Dinosaurs from the end of the Cretaceous remain poorly known in Africa and the Afro-Arabian plate, especially compared to the faunas of North America and Asia. Only a handful of localities, in Egypt^1–4^, Angola^5^, Oman^6^, and Morocco^7–10^ have produced Campanian—Maastrichtian dinosaurs. Initial finds suggested African dinosaurs resembled those of other Gondwanan landmasses, i.e. India, Madagascar, and South America, with faunas dominated by titanosaurians^1,9^ and abelisaurids^8^. However, the presence of hadrosauroids in the latest Cretaceous of Oman^6^ and Angola^5^ suggests a more complicated picture. More recently, a small hadrosaurid, *Ajnabia odysseus*^7^, was discovered in the late Maastrichtian phosphates of Morocco (Fig. 1).

Duckbill dinosaurs, or hadrosaurids, were highly successful herbivores that staged a major radiation in the Late Cretaceous^11–13^. Hadrosaurids evolved in North America in the Turonian^7^, before dispersing into Asia and Europe^7^. The presence of a hadrosaurid in Africa is perplexing, because Africa had been isolated from Laurasia by deep seaways since the mid-Jurassic^14^, while hadrosaurids evolved in the Late Cretaceous. The resolution to the paradox appears to be that duckbills swam or rafted to Africa^7^.

*Ajnabia* is related to the European lambeosaurines, Arenysaurini^7^, suggesting overwater dispersal from Europe. Yet with *Ajnabia* known by only a pair of maxillae and a partial dentary, our knowledge of African hadrosaurids is tantalizingly incomplete. We report three new hadrosaurid specimens from the late Maastrichtian of Morocco (Fig. 1). One represents a new small lambeosaurine, distinct from *Ajnabia*. Two specimens represent larger lambeosaurines, suggesting at least one additional species.

### Geographic and geological setting

The new hadrosaurid remains come from the marine phosphates of the Oulad Aboun Basin, in Khouribga Province. The new small lambeosaurine, *Minqaria bata*, comes from the mines at Sidi Chennane, the same locality that produced *Ajnabia odysseus*^7^, and is based on an associated skull. The other fossils come from Sidi Daoui and Mrah Lahrach (Fig. 1) and were found as isolated elements, as is typical of the phosphates.

**Figure 2:**
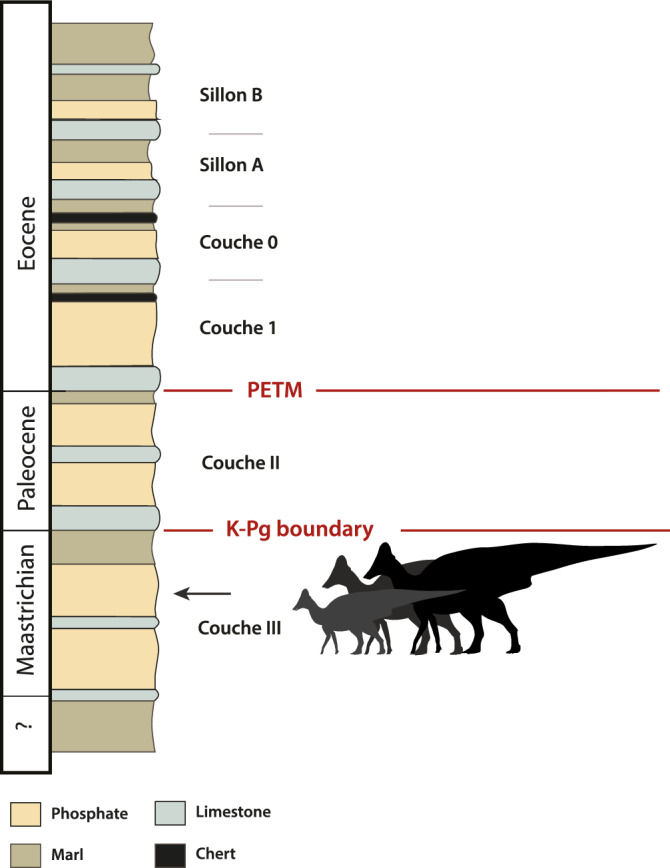
Synthetic stratigraphic column of the Phosphates of the Oulad Abdoun Basin of Morocco, showing position of hadrosaurid fossils in Upper Couche III, late Maastrichtian. After Kocsis et al.^15^.

The Phosphates consist of phosphatic sands, marls, and limestones (Fig. 2), deposited in an embayment along the eastern margin of the Atlantic from the late Cretaceous to the early Eocene^15,16^. The Phosphates are divided into beds or ‘couches’. Couche III is Cretaceous; Couche II, I and 0, are Paleogene (Danian—Ypresian). Couche III is Maastrichtian based on shark teeth^17,18^ and chemostratigraphy^15^, upper Couche III is latest Maastrichtian, dating to ~ 1 Ma or less before the K-Pg boundary.

Couche III is dominated by abundant and diverse marine vertebrates, and represents a highly productive upwelling zone^19^. Shark teeth^17,18^ and fish remains^17^ are the most common fossils. Marine reptiles are exceptionally common, and include diverse mosasauroids^17,20–33^, sea turtles, elasmosaurid plesiosaurs^34,35^, and rare crocodilians^36^; pterosaurs are also common^37,38^.

Dinosaurs are very rare as a proportion of fossils, but the exceptional abundance of fossils means Morocco’s Phosphates currently have the best record of dinosaurs from the latest Cretaceous of Africa. Dinosaurs include titanosaurians^9^, the abelisaurid *Chenanisaurus barbaricus*^8,10^ and two smaller abelisaurids^39^, and the lambeosaurine hadrosaurid *Ajnabia odysseus*^7^. Dinosaurs typically preserve as isolated bones^8,39^ and more rarely as associated remains^7,9^, a pattern also seen in the marine reptiles. This preservational mode likely results from carcasses spending time afloat, then falling apart due to decomposition and the action of scavengers; shark feeding traces are common on bones in the phosphates^29^.

### Systematic paleontology

Dinosauria Owen, 1842

Ornithischia Seeley, 1887

Iguanodontia, Baur, 1891

Hadrosauridae Cope, 1869

Lambeosaurinae Parks, 1923

Arenysaurini Longrich, Pereda-Suberbiola, Pyron et Jalil, 2021

*Minqaria bata* gen. et sp. nov.

**Etymology.** Arabic, ‘*minqar’*, beak; ‘*bata’*, duck.

**Figure 3:**
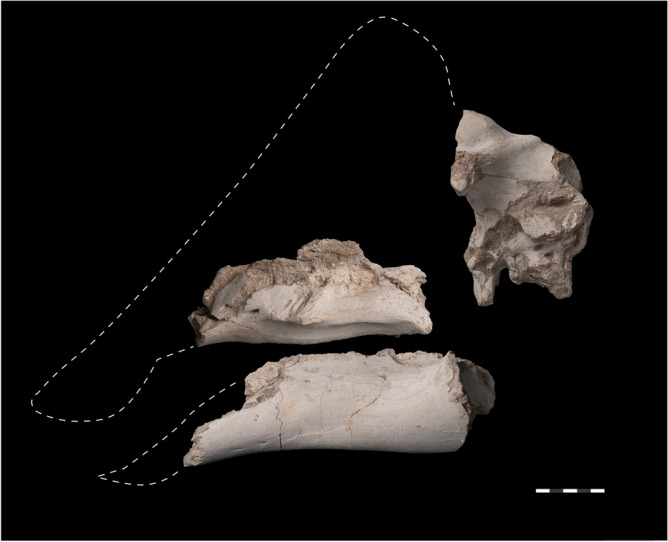
Skull elements of *Minqaria bata* nov. gen. et sp., MHNM.KHG.1395, holotype: right maxilla, braincase, left dentary. Sidi Chennane, Oulad Abdoun Basin, Upper Couche III, late Maastrichtian. Scale = 5 cm.

**Figure 4:**
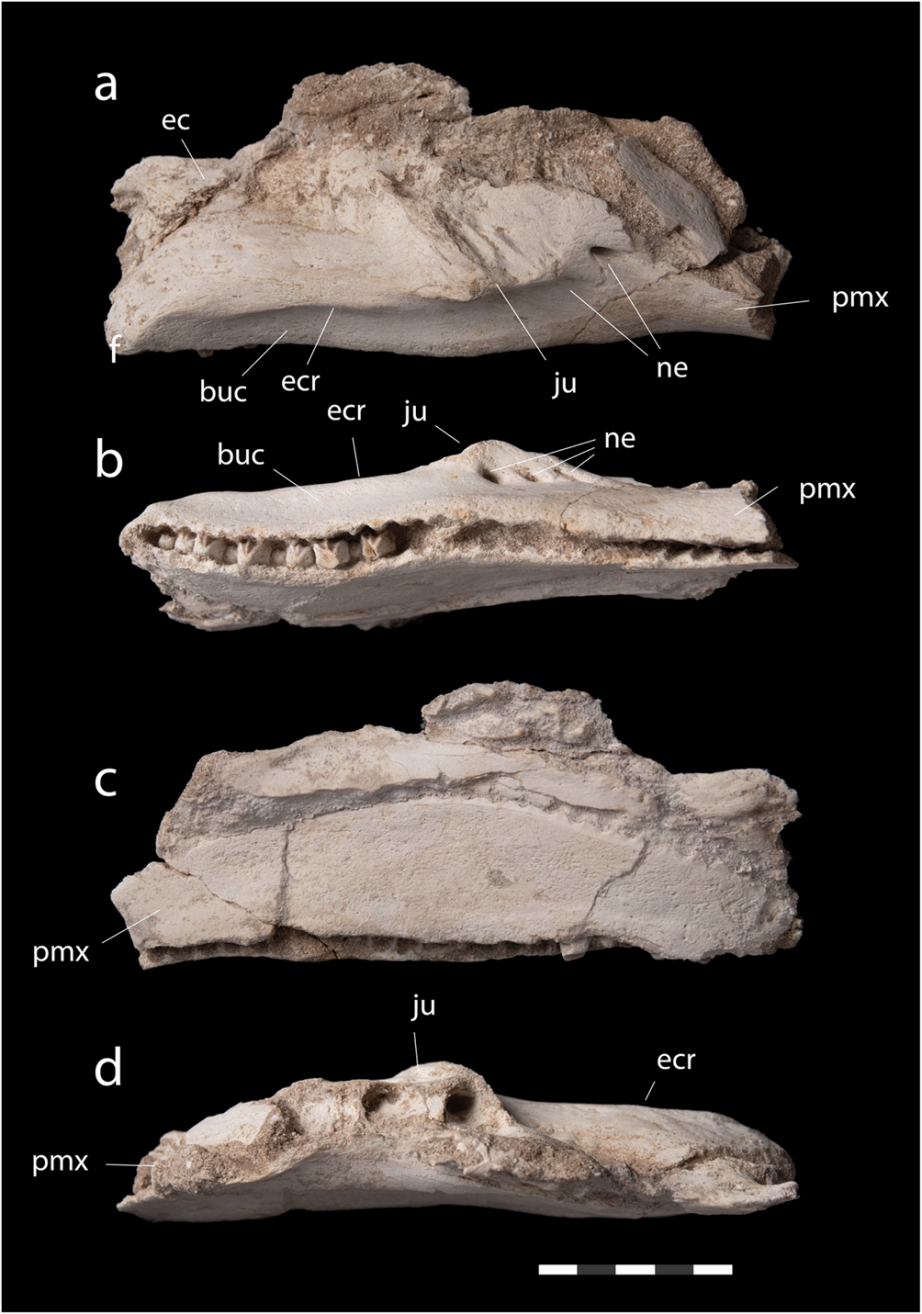
*Minqaria bata* nov. gen. et sp., MHNM.KHG.1395, holotype, Sidi Chennane, Oulad Abdoun Basin, Upper Couche III, late Maastrichtian. Right maxilla of in (**a**), lateral, (**b**), ventral, (**c**), medial, and (**d**), dorsal views. Abbreviations: buc, buccal fossa ec, ectopterygoid process, ecr, ectopterygoid ridge; ju, jugal articular facet, ne, neurovascular foramina, pmx, premaxillary process. Scale = 5 cm.

**Figure 5:**
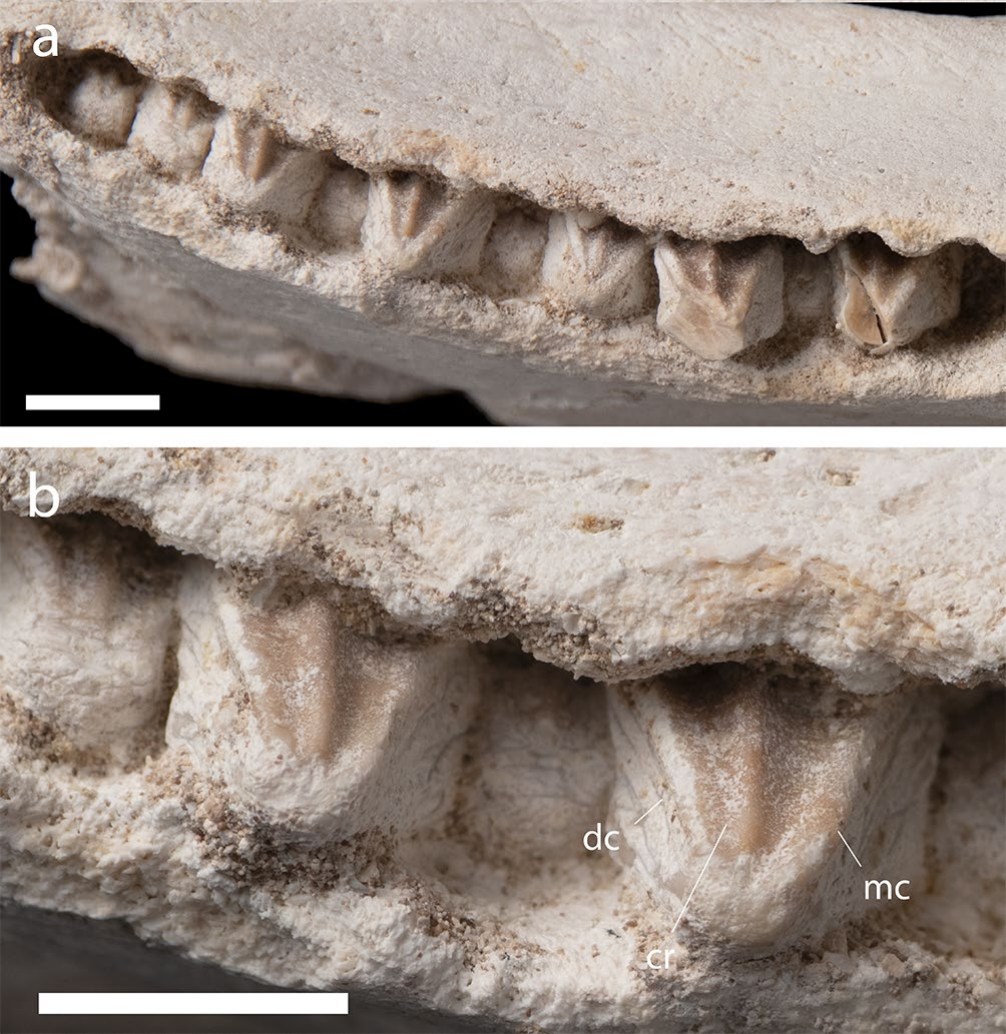
*Minqaria bata* nov. gen. et sp., MHNM.KHG.1395, holotype, Sidi Chennane, Oulad Abdoun Basin, Upper Couche III, late Maastrichtian. Dentition. (**a**), posterior maxillary dentition, (**b**), closeup of teeth. Abbreviations: cr, central ridge; dc, distal carina; mc, mesial carina. Scale = 1 cm.

**Figure 6:**
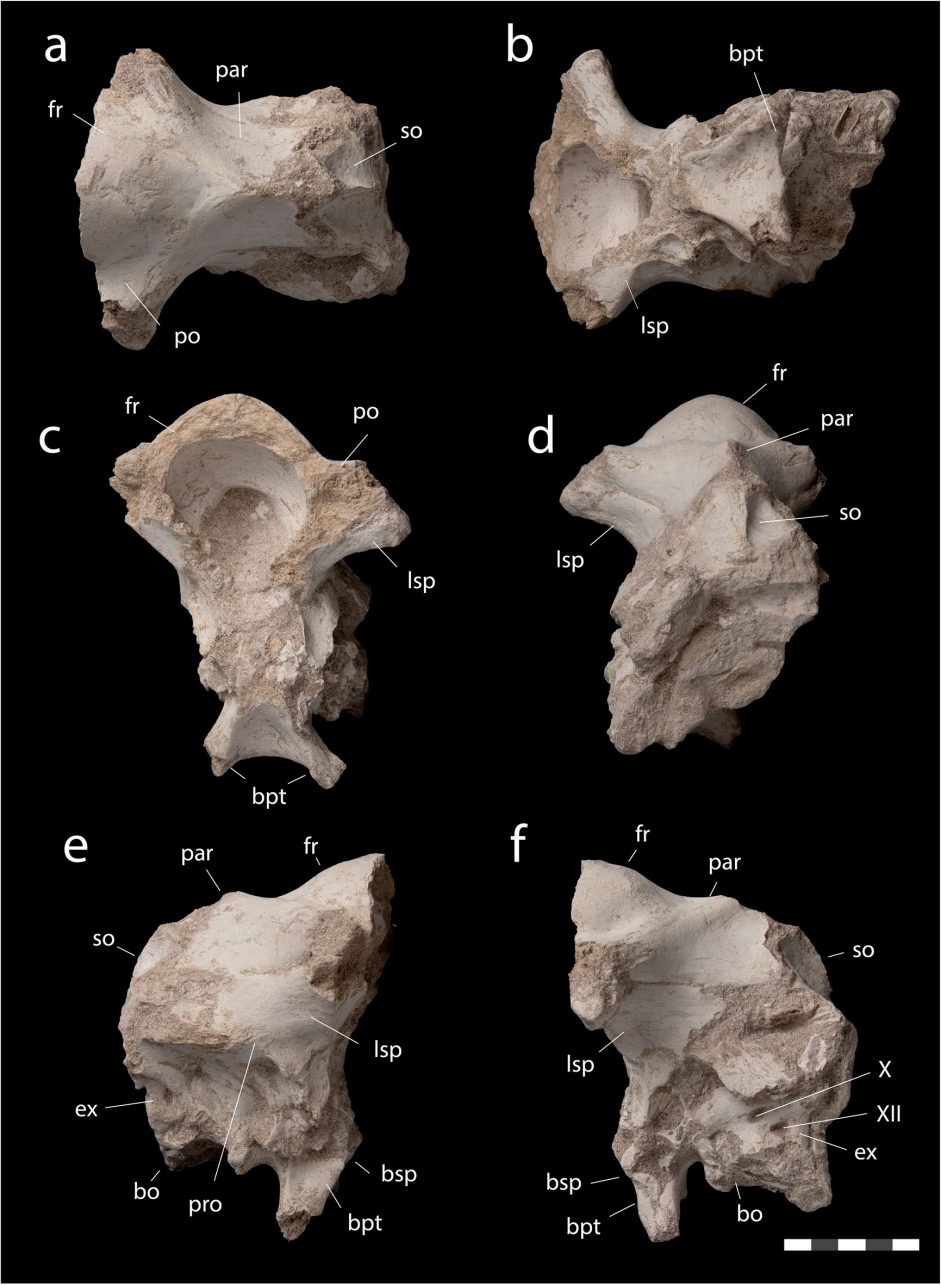
*Minqaria bata* nov. gen. et sp., MHNM.KHG.1395, holotype, Sidi Chennane, Oulad Abdoun Basin, Upper Couche III, late Maastrichtian. Braincase in (**a**), dorsal, (**b**), ventral, (**c**), anterior, (**d**), posterior, (**e**), right lateral, and (**f**), left lateral views. Abbreviations: *Bo*, basioccipital; *bpt*, basipterygoid processes; *Bsp*, basisphenoid; *Ex*, exoccipital, *Fr*, frontal; *Lsp*, laterosphenoid; *Par*, parietal; *Pro*, prootic; *So*, supraoccipital. Roman numerals (X, XII) = cranial nerve foramina. Scale = 5 cm.

**Figure 7:**
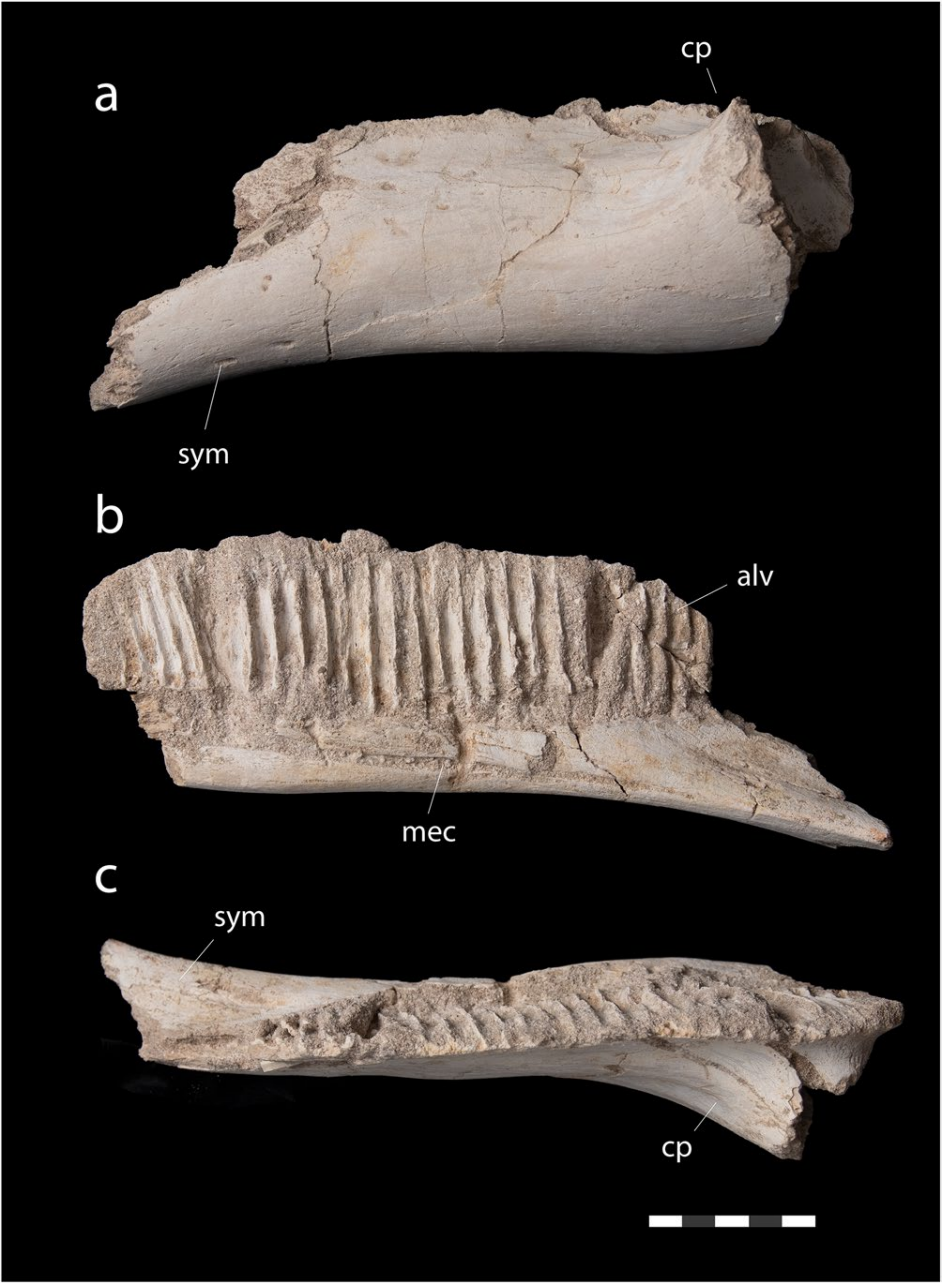
*Minqaria bata* nov. gen. et sp., MHNM.KHG.1395, holotype, Sidi Chennane, Oulad Abdoun Basin, Upper Couche III, late Maastrichtian. Dentary in (**a**), lateral, (**b**), medial, and (**c**), dorsal views. Abbreviations: *alv*, alveolar slots; *cp*, coronoid process; *mec*, Meckelian canal; *sym*, symphysis. Scale = 5 cm.

**Holotype.** MHNM.KHG.1395 (Museum d'Histoire Naturelle de Marrakech, Marrakech, Morocco, Cadi Ayyad University), right maxilla with teeth, left dentary, and braincase (Figs. 3, 4, 5, 6 and 7). The remains found in association by locals working the mines; color and preservation of the bone and associated matrix, size, and lack of duplication of elements are consistent with an associated individual.

**Locality and horizon.** Sidi Chennane, Oulad Abdoun Basin, Morocco; upper Couche III, late Maastrichtian (Figs. 1, 2).

**Diagnosis.** Small lambeosaurine characterized by the following character combination, which also differentiates it (where the specimens overlap) from *Ajnabia odysseus*. Jugal articulation lies very low on maxilla (autapomorphic within lambeosaurinae); ectopterygoid ridge ends at jugal articulation. Ectopterygoid ridge concave in lateral and dorsal views, narrower posteriorly than anteriorly. Neurovascular foramina arranged in a row. Highly domed frontoparietal, with extensive contribution of parietal to the dome, and a triangular parietal table. Maxillary toothrow sinusoidal in lateral or ventral views, with a deep buccal fossa. Dentary short and deep, occlusal margin straight; symphyseal process strongly extended anteriorly, and with straight ventral margin. Alveolar ridges of maxilla and dentary poorly developed. Teeth small, with narrow apices, broad central ridges, and rugose enamel.

### Description and comparisons.

*Maxilla*. The maxilla (Figs. 3, 4) is 195 mm long as preserved, but is incomplete anteriorly and dorsally. Alveoli become much smaller anteriorly (Fig. 4b), suggesting the maxilla did not extend much further; perhaps 15–20% of the bone’s length is missing anteriorly. There are 27 alveoli preserved and perhaps two or three on the missing part of the maxilla. This is low for Lambeosaurinae, which typically have more than thirty teeth^40–43^, but a low tooth count occurs in *Ajnabia odysseus*^7^ and *Canardia garonnensis*^44^. Because hadrosaurids added tooth positions as they grew^40^, this feature may result from the animal’s small size.

In lateral view (Fig. 4a), the maxilla is short anteroposteriorly and tall dorsoventrally, as in other hadrosaurids^11^ especially Lambeosaurinae^7,45^, and subrectangular. The maxilla’s anterodorsal margin extends up and forms a broad dorsal process, as in Saurolophinae^11^ and Arenysaurini including *Canardia garonnensis* and *Pararhabdodon isonensis*^44^; the maxilla is broken here in *Ajnabia*^7^. In Lambeosaurini^46^ and Parasaurolophini^42,45^, the maxilla is shallow anterior to the jugal articulation, with the premaxilla approaching the jugal articulation. The maxilla is narrow in dorsal (Fig. 4d) or ventral view (Fig. 4b), similar to *Pararhabdodon isonensis*; maxillae of *Ajnabia* and *Canardia* are broad in dorsal or ventral view.

The premaxillary process is downturned anteriorly (Fig. 4a), as in *Ajnabia*^7^ and other Arenysaurini^44^, and certain lambeosaurines such as *Corythosaurus casuarius*^47^, *Amurosaurus riabinini*^41^, *Olorotitan arharensis*^42^ and *Magnapaulia laticaudus*^43^. The maxilla’s anterolateral surface is convex; it is flatter in *Ajnabia*^7^, *Pararhabdodon*^44^, and *Canardia*^44^. The toothrow is sinusoidal in lateral view, curving inward anteriorly, outwards below the jugal facet, and curving in again posteriorly; the toothrow is straighter in lateral view in *Ajnabia*^7^ and the arenysaurins *Pararhabdodon*^44^ and *Canardia*^44^. The teeth are strongly inset medially (Fig. 4b), creating a deep buccal fossa absent in *Ajnabia*^7^ and other arenysaurins^44^.

The jugal facet lies very low on the maxilla (Fig. 4a), unlike all other Lambeosaurinae, where the jugal facet lies high on the maxilla^7,45^, an autapomorphy of the species. Here *Minqaria* resembles saurolophines such as *Kritosaurus* and *Edmontosaurus*^45^. The jugal articulation is convex anteroventrally, then forms a straight edge where it extends above the ectopterygoid ridge. Extension of the jugal articulation posterodorsally above the ectopterygoid ridge is an apomorphy of Lambeosaurinae^7,45^.

Five neurovascular foramina extend in a line from below the jugal articulation anteriorly onto the maxilla’s lateral surface (Figs. 4a, b). This differs from *Ajnabia*^7^, where a pair of neurovascular foramina lie below the jugal articulation. Neither is this condition seen in *Pararhabdodon*^44^, but it is similar to *Canardia*^44^.

The ectopterygoid ridge (Fig. 4a) extends from the jugal articulation back along the maxilla’s posterior dentigerous. It forms a low, broad ridge, similar to that of *Ajnabia*^7^; the ectopterygoid ridge in *Canardia* and *Pararhabdodon* is narrower and better defined^44^. The ridge has a distinctly concave ventral margin, similar to *Canardia*, those of *Ajnabia* and *Pararhabdodon* are straight in lateral view^44^. In dorsal view (Fig. 4d), the shelf formed by the ectopterygoid ridge narrows towards the back of the jaw; that of *Ajnabia*^7^ becomes broader, and is more convex.

In ventral view (Fig. 4b), the toothrow is sinusoidal. The toothrow curves inwards at the front of the jaw, outwards near the middle of the maxilla, then curves inwards again at the back. *Ajnabia*^7^ has a similar but less extreme curvature, the toothrow of *Pararhabdodon* is only weakly sinusoidal^48^. The toothrow in *Canardia* is strongly curved outward posteriorly^44^, but this feature may be exaggerated by crushing.

*Maxillary dentition*. Several unerupted teeth lie partially exposed in the maxillae (Figs. 4, 5). These teeth are typical of hadrosaurids in being much taller than wide^11^ and resemble lambeosaurines^41,46,49–51^ in their tall, narrow, lanceolate shape (Fig. 5). The tips of the crowns are narrower and more pointed than in *Ajnabia odysseus*^7^. As in other hadrosaurids, maxillary teeth bear enamel on the lateral surface of the tooth only^11^. The enamel has a distinctly coarse, roughened texture, *Ajnabia*^7^ has smoother enamel.

The mesial and distal carinae have highly reduced denticles, a derived feature shared with other Arenysaurini^7^, but unlike most Lambeosaurinae^11^ where denticles are large and well-developed. The single central ridge is slightly offset towards the distal carina, as in *Ajnabia*^7^. The central ridge is low and rounded; in *Ajnabia* it is taller and narrower. The lateral, enamel-covered surface of the tooth is not planar, but instead curls onto the apex of the tooth, as in *Ajnabia*^7^.

*Braincase*. The braincase (Fig. 6) comprises the frontals, parietal, orbitosphenoids, laterosphenoids, prootics, supraoccipital, basisphenoid, basioccipital, and exoccipitals. Overall, the braincase closely resembles that of *Arenysaurus*^11^.

The frontals contact each other medially, the parietal posteriorly (Fig. 6a), the laterosphenoid ventrally. Although the sutures are visible, the frontals are tightly joined to each other and the parietal by a strongly interdigitating contact, similar to that seen in other lambeosaurines^52^. The degree of interdigitation is stronger than in *Arenysaurus*^50^, but not developed to the same degree as seen in *Corythosaurus*^52^. In places the frontoparietal suture is well-developed, but in others is difficult to see, due either to tight knitting of bones or perhaps partial fusion of bones. The tight knitting of the bones suggests the animal is mature.

The frontals form a well-developed dome, as in *Arenysaurus*^50^, although the frontals of *Minqaria* are less strongly domed than in *Arenysaurus*, where the posterior margin of the frontals rises up almost vertically. A well-developed dome also occurs in *Jaxartosaurus*. The frontals of Lambeosaurini^40^ and Parasaurolophini^53,54^are less strongly domed than in *Minqaria* or *Arenysaurus*. This may be partly size-related, as juvenile lambeosaurines, e.g. *Hypacrosaurus*, show stronger frontal domes than adults^40^.

Laterally, frontal postorbital processes are slightly concave. Posteriorly, the frontals form a V-shaped notch to receive a triangular tab from the parietal.

The parietal contacts the frontals anteriorly (Fig. 6a), the laterosphenoids anteroventrally, and the supraoccipital posteriorly (Fig. 6e, f). Along the midline the parietal forms a broad, triangular anterior process projecting between the frontals, contributing to the doming of the skull roof. A similar morphology is seen in *Arenysaurus*^50^; in *Jaxartosaurus* the parietal has a broad, quadrangular contribution to the dome. A similar triangular process contributing to the frontoparietal dome occurs in juvenile *Corythosaurus* but is reduced in adults^52^. In the basal hadrosauroid *Eotrachodon*, a triangular process projects between the frontals^55^, but does not contribute to the dome; the saurolophines *Edmontosaurus*^56^ and *Gryposaurus*^52^ lack this triangular process.

The parietal’s anterolateral processes angle forward and wrap around the back of the frontals. A similar morphology occurs in *Arenysaurus*, *Jaxartosaurus*, the lambeosaurin *Corythosaurus*^54^ and the parasaurolophin *Charonosaurus*. The anterolateral processes project laterally in Saurolophinae^52,56^ and *Eotrachodon*^55^. The parietal has a small, triangular parietal table and behind this a parietal sagittal crest; in *Arenysaurus*^50^ the parietal lacks a parietal table and the sagittal crest extends further forward towards the frontals.

The parietal is broadly arched between the supratemporal fenestrae, with a narrow sagittal crest as in *Hypacrosaurus*^40^; the parietal of *Arenysaurus* is narrower^50^. The parietal is moderately elongate, resembling *Arenysaurus*, Lambeosaurini^52^ and Parasaurolophini^54^; the parietal of *Tsintaosaurus* is shorter; the parietal of Saurolophinae tends to be more elongate^52,56^. Ventrolaterally, the parietal curls down to contact the orbitosphenoids, laterosphenoids, and prootics.

Laterally (Fig. 6e, f), the laterosphenoid and prootic form the lateral walls of the braincase. Ventrally, the basisphenoid projects down forming a pair of long basipterygoid processes. The basipterygoid processes project caudally as in *Arenysaurus*^50^ and *Olorotitan*^42^; the basipterygoid processes project ventrally in *Hypacrosaurus altispinus*^40^ and the saurolophines *Brachylophosaurus canadensis*^57^, and *Edmontosaurus regalis*^56^ and anteroventrally in *Parasaurolophus cyrtocristatus*^53^.

Posterior to the basipterygoid processes, the basisphenoid is markedly concave, as in *Arenysaurus*^11^. The basisphenoid’s alar process is well-developed and posterolaterally projecting. It is similarly well-developed in *Arenysaurus*, but projects more laterally. The alar process is small in most hadrosaurids except brachylophosaurins^57^. Posteriorly, the supraoccipital is tall and triangular, with a midline crest not seen in other hadrosaurids^40,54,56^. The foramen magnum and occipital condyle are obscured by hard matrix. Two cranial nerves perforate the exoccipital, (Fig. 6f) forming the exits of the hypoglossal (XII) nerve posteriorly and of the glossopharyngeal and vagoaccessory nerves (IX-XI) anteriorly through what appears to be a single metotic foramen (Fig. 6f).

*Dentary*. The dentary (Fig. 7) is short and deep, similar to *Blasisaurus canudoi*^49^, *Corythosaurus casuarius*^47^ or *Hypacrosaurus altispinus*^40^, and unlike the elongate dentary of the arenysaurins *Arenysaurus ardevoli*^50^ and *Koutalisaurus kohlerorum*^58^, or lambeosaurines such as *Amurosaurus riabinini*^41^, *Olorititan arharensis*^42^, *Sahaliyania elunchunorum*^59^, and *Parasaurolophus tubicen*^51^. The dentary’s dorsal and ventral margins are roughly parallel in lateral view (Fig. 7a), giving it a rectangular shape. The dentary’s dentigerous margin is straight for most of its length, but turns downwards at the anterior and posterior end of the toothrow, similar to *Blasisaurus*^49^ and *Koutalisaurus*^58^; the dentigerous margin of *Arenysaurus*^9^ is more convex. The dentary’s ventral margin is nearly straight, with a slight inflection where the symphyseal process projects anteroventrally. This weak inflection and the anteriorly projecting symphyseal process are shared with *Koutalisaurus*^58^; in *Arenysaurus*^50^ and *Blasisaurus*^49^, the ventral margin of the jaw curves more and the symphyseal process projects more strongly downward. The posteroventral margin of the dentary is weakly convex. The coronoid process is strongly projected laterally as in other hadrosaurids.

In medial view (Fig. 7b), there are at least 25 alveoli, formed as long, narrow grooves to accommodate multiple replacement teeth, as in other Hadrosauridae^11^. Alveolar slots are shallower than in *Ajnabia*^7^. Alveoli extend three-quarters of the way down the dentary. Alveoli extend further ventrally in *Blasisaurus*^49^, *Koutalisaurus*^58^, and *Hypacrosaurus*^40^, and almost all the way down in *Arenysaurus*^50^. The Meckelian canal is visible medially as in *Blasisaurus* and *Koutalisaurus*^58^; it runs ventrally and is concealed in medial view in *Arenysaurus*^50^ and *Hypacrosaurus*^40^. In dorsal view (Fig. 7c), the dentary is narrow and the toothrow has a sinuous curve matching the curve of the maxillary toothrow.

Lambeosaurinae indet.

**Figure 8:**
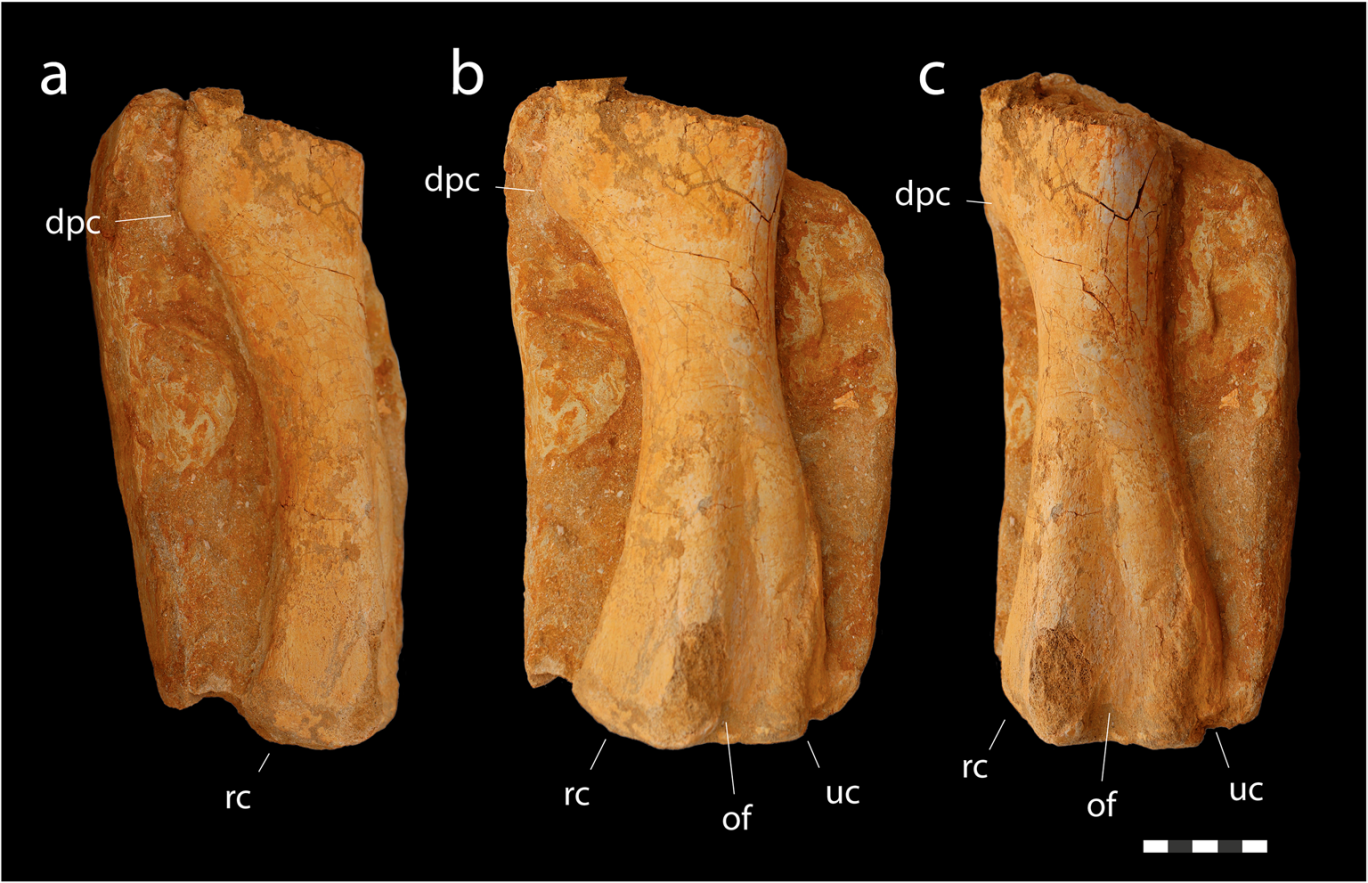
Lambeosaurinae indet., MHNM.KHG.1483, Sidi Daoui, Oulad Abdoun Basin, Upper Couche III, late Maastrichtian. Left humerus in posterior (**a**), posterolateral (**b**) and lateral (**c**) views. Abbreviations: *dpc*, deltopectoral crest; *of*, olecranon fossa; *rc*, radial condyle; *uc*, ulnar condyle. Scale = 5 cm.

**Figure 9:**
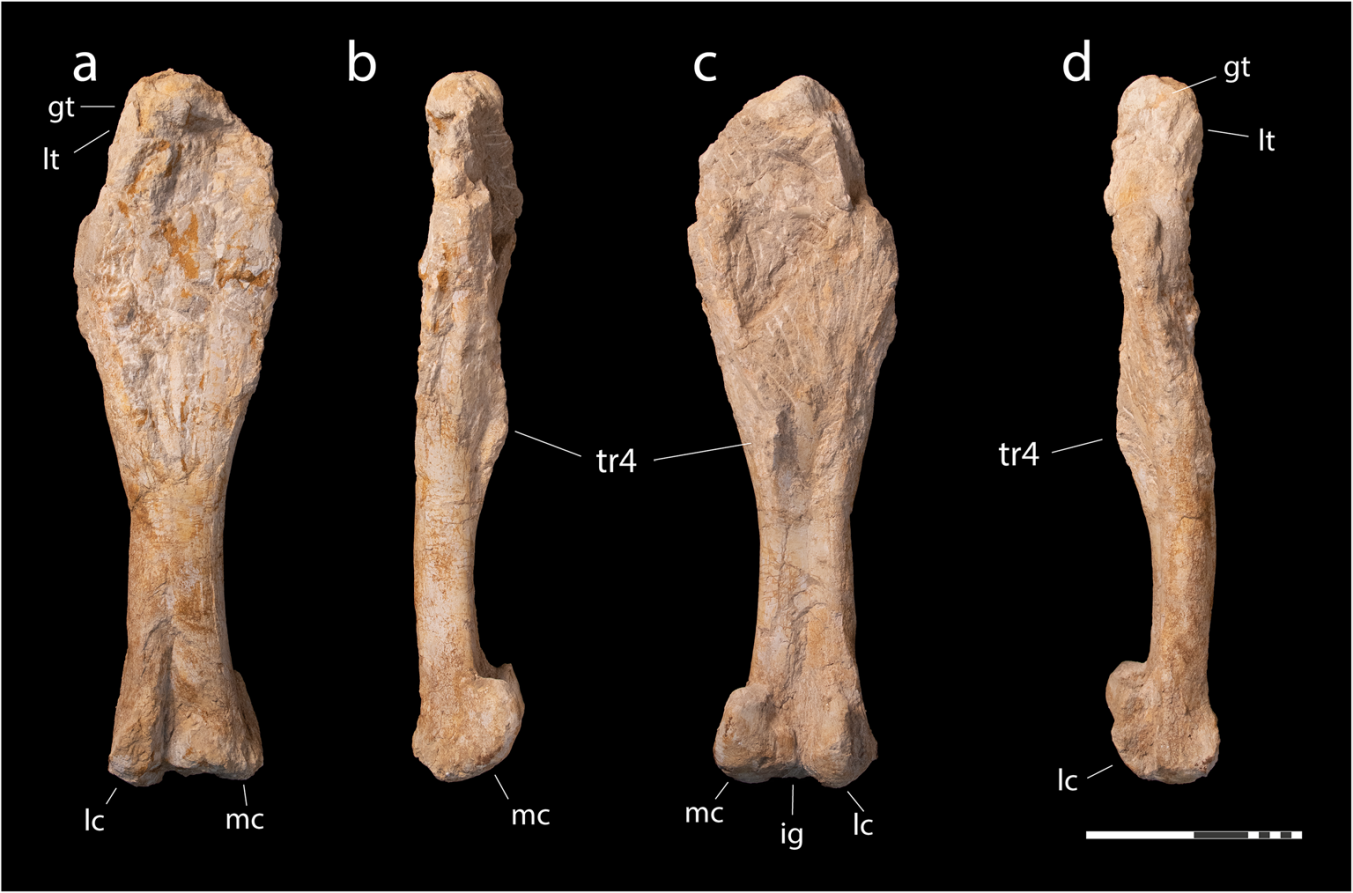
Lambeosaurinae indet., MHNM.KHG.1484, Mrah Lahrach, Oulad Abdoun Basin, Upper Couche III, late Maastrichtian. Right femur in anterior (**a**), medial (**b**), posterior (**c**), and lateral (**d**) views. Abbreviations: *gt*, greater trochanter; *ig*, intercondylar groove; *lc*, lateral condyle, *lt*, lesser trochanter; *mc*, medial condyle; *tr4*, fourth trochanter. Scale = 20 cm.

*Material*. MHNM.KHG.1483, left humerus (Fig. 8); MHNM.KHG.1484, right femur (Fig. 9).

*Locality and Horizon*. MHNM.KHG.1483 comes from Sidi Daoui, and MHNM.KHG.1484 from Mrah Lahrach, in the Oulad Abdoun Basin of Morocco (Fig. 1). Both come from upper Couche III, corresponding to the late Maastrichtian (Fig. 2).

### Description and comparisons

*Humerus*. MHNM.KHG.1483 is a left humerus missing its proximal end (Fig. 8). The bone measures 278 mm long, and likely measured around 435 mm when complete. Assuming proportions similar to *Corythosaurus casuarius*^60^, MHNM.KHG.1483 was around 5.9 m in length.

In lateral view (Fig. 8a), the humerus is long and gracile. It has a weak sigmoidal curvature, the shaft being bowed anteriorly at the level of the deltopectoral crest, and the distal shaft being bowed posteriorly. The distal shaft is weakly curved, as in other lambeosaurines^54^, including the arenysaurins *Canardia garonnensis*^44^ and cf. *Pararhabdodon isonensis*^61^; the distal shaft is more strongly curved in Saurolophinae^62,63^, but straight in some basal hadrosauroids^64^.

In posterior view (Fig. 8c), the humerus is narrow at midshaft and broadly flared distally. The radial and ulnar condyles are well-developed. A deep olecranon fossa separates the radial and ulnar condyles, and extends about halfway up the shaft.

The prominent deltopectoral crest (Fig. 8b) projects strongly anterolaterally. The distal margin is strongly concave as in other lambeosaurines^44,54^ and saurolophines^62,63^, but unlike *Hadrosaurus foulkii*^65^ or basal hadrosauroids^64,66^ where it is weakly concave, or straight.

*Femur*. MHNM.KH.1484 (Fig. 9) is a right femur. It measures 640 mm in length, with a midshaft circumference of 238 mm. Scaling a skeletal reconstruction of *Corythosaurus*^60^ to a femur length of 640 mm suggests a length of ~ 4.5 m.

The femur’s proximal end is crushed (Fig. 9a), and the bone is difficult to differentiate or physically separate from the consolidated matrix, complicating comparisons. The shaft is large in diameter despite the animal’s small size. It is anteroposteriorly crushed but even accounting for crushing, it is robust, even compared to large hadrosaurids like *Magnapaulia laticaudus*^43^ and *Olorotitan arharensis*^42^.

The femoral shaft appears to be relatively straight in anterior view (Fig. 9a), as in Lambeosaurinae^43,67^, Edmontosaurini^63^, and Hadrosaurini^65^. In basal hadrosauroids, the femoral shaft is bowed^66,68^ with the condyles more obliquely oriented relative to the femoral shaft, suggesting a less vertical femoral posture.

In medial or lateral view (Fig. 9b, d), the shaft has a gentle sigmoidal curvature, with the proximal shaft being bowed posteriorly, and the distal end bowed anteriorly. This sigmoid curvature occurs in the type of *Orthomerus dolloi*^68^, but not in other specimens^69^, and in Parasaurolophini^54^, but not in Lambeosaurini^43^. The femur of the arenysaurin *Adynomosaurus arcanus* is straight in lateral view^67^, but has also been reconstructed. The femoral shaft is also straight in some saurolophines, e.g. *Secernosaurus koerneri*^70^ and *Saurolophus osborni*^71^. Sigmoidal curvature of the femoral shaft occurs in some basal hadrosauroids, including *Bactrosaurus johnsoni*^72^ and *Telmatosaurus transsylvanicus*^68^, but it is straighter in others, such as *Gobihadros mongoliensis*^64^.

There is a convex fourth trochanter at midshaft (Fig. 9b), on the posteromedial surface of the shaft. The fourth trochanter is weakly projecting, shared with the arenysaurin *Adynomosaurus arcanus*^67^, the Basturs Poble arenysaur^61^, a femur from the Maastricht Formation of the Netherlands^73^, and *Orthomerus dolloi*^68,69^. The fourth trochanter is a prominent, winglike triangular structure in parasaurolophins^42^, lambeosaurins^43^, saurolophins^74^, and basal hadrosauroids^64,68,72^. A low fourth trochanter may be a diagnostic feature of Arenysaurini.

Medial and lateral condyles are well-developed and project anterior and posterior to the shaft as in other hadrosaurids. The cranial intercondylar groove is broadly open (Fig. 9c). There is a deep posterior intercondylar groove. Because body mass is tightly correlated with limb bone dimensions, particularly diameters and circumferences^75^, it is possible to make a mass estimate from the femur. Given a femur circumference of 238 mm and assuming a ratio of humerus circumference to femur circumference of 0.651, as seen in *Corythosaurus*^76^, the corresponding humeral circumference should be 154.9. A regression of humerus + femoral circumference suggests a mass of 1066 kg based on the equation for quadrupeds from Campione and Evans^75^ and 1099 kg for the equation for quadrupeds from Campione 2017^77^ for this ~ 4.5 m long animal. The Sidi Daoui humerus suggests a larger, 5.9 m long animal, approximately twice this mass, but smaller than contemporary Laurasian hadrosaurids.

## Discussion

*Ontogeny.* Skeletal fusion can be used as a proxy for maturity in dinosaurs. Although some elements (e.g. parietals) can fuse early in ontogeny, extensive skull fusion occurs as animals approach full size^40,78,79^. Extensive co-ossification of the braincase in the *Minqaria* holotype is therefore consistent with skeletal maturity. No clear exoccipital-prootic suture is visible, suggesting these elements are fused; fusion between these bones occurs late in ontogeny in lambeosaurines^40^. The prootic-laterosphenoid suture is also indistinct suggesting fusion; the laterosphenoid—parietal suture is visible anteriorly but not posteriorly, suggesting partial fusion. This extensive cranial fusion contrasts with juvenile and subadult lambeosaurines^40,80^, showing MHNM.KH.1484 is a mature adult.

Frontal-frontal and frontal-parietal sutures are also highly interdigitated and the midline of the parietal forms a sagittal crest, as in adult lambeosaurines^40,81^. Finally, surface texture of the bone suggests maturity. Juvenile lambeosaurines, like other juvenile dinosaurs, show striated bone textures associated with rapid growth^82^; but the bone of MHNM.KH.1484 lacks striated texture.

*Systematics. Minqaria is* distinguished from *Ajnabia* by the shape of the maxilla (Fig. 4), which has a more ventrally placed jugal facet, a curved ectopterygoid ridge, a more sinusoidal toothrow, and neurovascular foramina arranged in a line. However, *Minqaria* closely resembles *Ajnabia* and other arenysaurins in its small size, and many anatomical features. Derived features uniting *Minqaria* with arenysaurins include the reduced number of maxillary alveoli (shared with *Ajnabia* and *Canardia*), a posteriorly shifted dental midline ridge (shared with *Ajnabia*, *Blasisaurus*, and the Serrat del Rostiar lambeosaurine); a strongly domed frontal (shared with *Arenysaurus*, and the Basturs lambeosaurine). The dentary predentary process appears to resemble that of other arenysaurins in being triangular, with straight dorsal and ventral margins, although damage makes comparisons difficult. Accordingly, *Minqaria* is recovered within Arenysaurini, a lambeosaurine clade endemic to Europe and North Africa.

The humerus and femur appear to represent lambeosaurines. The humerus resembles Hadrosauridae, including Lambeosaurinae, in having a concave distal edge to the deltopectoral crest; the weak sigmoidal curvature of the shaft resembles Lambeosaurinae. Neither is unique to Lambeosaurinae, but this character combination seems to be unique to lambeosaurines. The femur resembles Hadrosauridae in having a straight shaft in anterior view. The sigmoidal curvature seen in lateral view is seen in a variety of taxa, but is consistent with referral to Lambeosaurinae. The long, low fourth trochanter suggests affinities with Arenysaurini. Neither the humerus nor femur are diagnostic, and are referred to Lambeosaurinae indet. Given their large size, they are likely distinct from either *Ajnabia* or *Minqaria*, but additional fossils are required to test this hypothesis.

### Phylogeny and biogeography

Although the lambeosaurine affinities of the Moroccan hadrosaur *Ajnabia* have been questioned^83^, the new material corroborates the presence of Lambeosaurinae in Africa. Other analyses have recovered *Ajnabia* as a lambeosaurine^84,85^ but not a monophyletic clade of European and African species. The differences result from inclusion of novel characters (e.g. the triangular predentary process of the mandible) and recodings of characters of the jaws and teeth that were found to unite Arenysaurini. We argue the results found here- with small-bodied European and African lambeosaurines representing a single lineage- are more parsimonious than the alternatives, which require multiple overwater dispersals to colonize Iberia, long ghost-lineages, and repeated evolution of small body size. The simplest explanation of all the data is that European and African lambeosaurines represent one lineage. More complete remains of the African and European arenysaurins are needed to better resolve their systematics, however.

**Figure 10:**
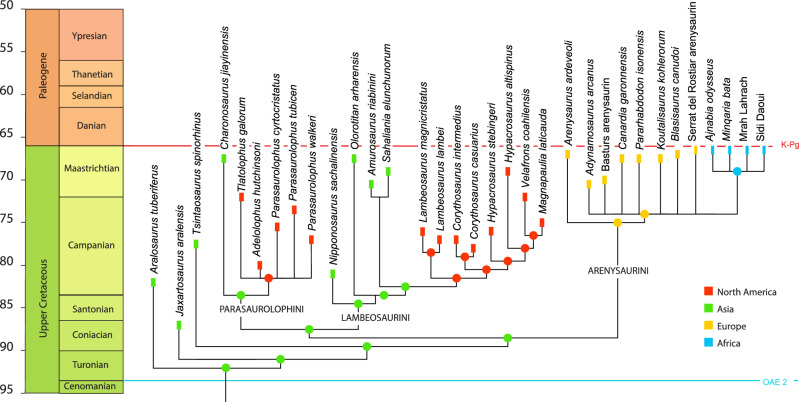
Evolutionary tree of Lambeosaurinae. European and African lambeosaurines are placed in Arenysaurini, a basally-diverging group of lambeosaurines.

Our phylogenetic analysis suggests Lambeosaurinae initially diversified in Asia, then saw dispersals into North America by Parasaurolophini and Lambeosaurini. Dispersal from Asia into Europe was followed by dispersal into North Africa. Multiple dispersals from Europe to Africa seem less parsimonious than a single dispersal (Fig. 10), but are not impossible, particularly given the distinctive morphologies of *Ajnabia* and *Minqaria*.

The precise number of dispersals in hadrosauroids remains unclear due to conflicting tree topologies. The existence of basal hadrosauroids and lambeosaurines in Europe suggests at least two dispersals^7^, followed by dispersal of lambeosaurines into Africa^7^. The discovery of basal hadrosauroids in South America suggests two dispersals into South America, one by basal hadrosauroids^83^ and one by kritosaurins^86,87^, which dispersed into Antarctica^86^. Hadrosaurines dispersed into Appalachia; reinterpretation of *Lophorhothon* as a basal hadrosauroid suggests saurolophines did not disperse into Appalachia^88^. Absent any strong evidence for land bridges, and given the limited number of dispersals of dinosaurs between these land masses, the simplest hypothesis is that hadrosauroids colonized Europe, Africa, Appalachia, South America and Antarctica via oceanic dispersal^7^.

**Figure 11:**
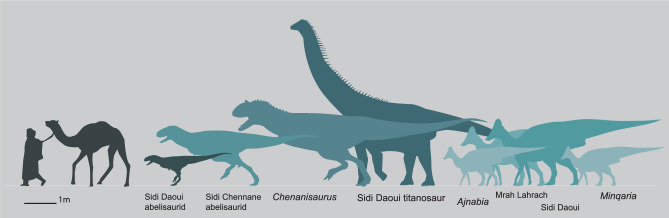
Late Maastrichtian dinosaurs of the latest Cretaceous Phosphates of Morocco.

*Diversity of North African lambeosaurines*. The discovery of *Ajnabia* was surprising given that, during the Cretaceous, hundreds of kilometers of water separated North Africa from Eurasia. The new lambeosaurine fossils not only confirm the existence of lambeosaurines in North Africa, but shows they were diverse. Although *Minqaria* is similar in size to *Ajnabia*, differences in the shape of the maxilla, the position of the jugal contact, and especially the tooth morphology suggest that it likely occupied a distinct ecological niche. Meanwhile, the relatively larger size of the Daoui and Mrah Lahrach lambeosaurines suggests they occupied a niche distinct from the smaller *Ajnabia* and *Minqaria*. The different morphologies and sizes (Fig. 11) suggest that we are sampling a diverse radiation (Fig. 10). The relatively robust femur of the Mrah Lahrach animal is also curious suggesting an unusual locomotor strategy and perhaps ecology.

Given the long distance and deep ocean channels separating North Africa from Europe^89^, a single dispersal seems more likely than multiple dispersals. If so, Lambeosaurinae may have arrived in the Maastrichtian or Campanian, possibly during the Campanian–Maastrichtian lowstand event, then diversified over several million years. A possible hadrosaurid from the early Maastrichtian of Angola would potentially constrain lambeosaurine dispersal to the early Maastrichtian or late Campanian^5^, if it does represent a hadrosaurid. Given the rapid rates of speciation seen in colonizing lineages such as Galapagos finches^90^ and island anoles^91^, and high rates of turnover seen in herbivorous dinosaurs^92,93^ lambeosaurines could have produced a high diversity of species in just a few million years. Few other assemblages have a comparably high diversity of lambeosaurines, the exception being the Dinosaur Park Formation of Alberta, which for a brief interval contained the genera *Lambeosaurus*, *Corythosaurus*, and *Parasaurolophus*^93^, but what is remarkable about Morocco is that it shows high diversity despite far more limited sampling, suggesting that it draws from a highly diverse fauna.

Absence of competing ornithischians may have driven diversification. In contrast to North America or Asia, where hadrosaurids and ornithischians were highly diverse, no ornithischians other than lambeosaurines are known from the Maastrichtian of Morocco. Similarly, diversity of the European Lambeosaurinae may be high, with a wide range of jaw morphologies and species occurring, because hadrosaurids in Europe had limited competition, with relatively few other ornithischian lineages present. These included rhabdodontid iguanodontians^94^, basal hadrosauroids^95–97^, and struthiosaurine ankylosaurs^98,99^. North American lambeosaurines competed with multiple lineages of hadrosaurids as well as other ornithischians such as thescelosaurids, pachycephalosaurids, leptoceratopsids, nodosaurids and ankylosaurids, as well as herbivorous coelurosaurs such as ornithomimids, deinocheirids, caenagnathids, and possibly troodontids^100^.

By the latest Maastrichtian, lambeosaurines saw regional extinction in North America, disappearing from the northern Great Plains, but persisting in the south^101^. Meanwhile, lambeosaurines in Europe and especially the Ibero-Armorican landmass became highly diverse at the end of the Cretaceous^44,102,103^. A comparable diversification appears to have taken place in North Africa. These patterns emphasize the highly local nature of dinosaur diversity; local extinctions or radiations may not capture global patterns in dinosaur diversification and extinction.

The new fossils confirm that African hadrosaurids were small by hadrosaurid standards. The femur suggests an animal around 4.5 m in length and weighing ~ 1000 kg; the humerus suggests a substantially larger animal. This is large by the standards of extant mammals, but small by the standards of hadrosaurids. *Minqaria* was a smaller animal, around 3.5 m in length, which suggests a weight of around 250 kg, but the fusion of the braincase indicates it was somatically mature. *Ajnabia* was likely similar in size given the mature bone texture of the holotype.

Iberian arenysaurins resembled African forms in being relatively small. Small species include indeterminate species from Lleida^61,104^ falling within the size range of *Minqaria* and *Ajnabia*. In addition, very small lambeosaurine remains belonging to mature individuals of about 2 m body length have been described from latest Cretaceous sites of the Spanish Pyrenees^105^. The Basturs Poble lambeosaurine is somewhat larger, with a femur length of 645 mm, comparable to the larger hadrosaurids known from Morocco.

Meanwhile, in North America and Asia, hadrosaurids evolved large and giant forms among Lambeosaurini^43^, Parasaurolophini, Edmontosaurini^106^, and Kritosaurini^74,107^. The lambeosaurin *Corythosaurus* weighed ~ 3.5 tons and the giant *Magnapaulia* grew significantly larger; the parasaurolophin *Parasaurolophus walkeri* exceeded 5 tons^76^; *Gryposaurus latidens* reached 4 tons^76^, and a late Maastrichtian gryposaur from the Naashoibito shows some kritosaurs grew larger. *Saurolophus osborni* approached 7 tons^76^; a related Hell Creek form grew larger^108^. The edmontosaurin *Edmontosaurus* reached almost 8 tons^76^; in Asia, *Shantungosaurus* reached 17 tons^76^. Strikingly, small-bodied hadrosaurids (< 1000 kg) are unknown from latest Cretaceous North America and Asia.

While hadrosaurids in North America and Asia occupied megaherbivore niches, Moroccan and European lambeosaurines occupied small- and medium-sized herbivore niches. These patterns might result from the low diversity of competing small herbivores, and titanosaurian sauropods outcompeting lambeosaurines for megaherbivore niches.

Curiously, ornithischians occur in the latest Cretaceous of East Africa and Oman but do not appear to represent lambeosaurines. Ornithopods found in the Maastrichtian of Kenya represent non-hadrosaurid ornithopods (J. Sertich, pers. comm. 2023). Likewise, hadrosauroids from Oman^6^ represent non-hadrosaurids (D. Baastians, pers. comm. 2023). That lambeosaurines are (so far) unknown from East Africa or the Arabian plate could result from geographic barriers to dispersal. It is conceivable that the Trans-Saharan Seaway may have divided Africa into a series of smaller landmasses^39^, with endemic dinosaur faunas, but sampling remains and issue and more dinosaur fossils are needed from Africa to test this hypothesis.

## Conclusions

A hadrosaurid from the latest Maastrichtian phosphates of Sidi Chennane, Morocco, is distinct from *Ajnabia odysseus* and represents a new arenysaurin, *Minqaria bata*. *Minqaria* differs from *Ajnabia* in jaw and tooth morphology, suggesting it occupied a distinct niche. Fusion of cranial elements shows that it was mature despite its small size (~ 3.5 m), confirming the existence of small hadrosaurids in North Africa. Similarities with the European *Arenysaurus* provide further evidence for dispersal of lambeosaurines between the Ibero-Armorican landmass and Africa. A humerus from Sidi Daoui and a femur from Mrah Lahrach belong to larger individuals, suggesting at least three hadrosaur species occur in the phosphates. Even as lambeosaurines declined in the Maastrichtian of North America, they diversified in Africa.

## Methods

Phylogenetic analysis was conducted using a modified version of the character-taxon matrix from Longrich et al.^7^, which is derived from the matrix Kobayashi et al.^63^. Characters are from Xing et al.^109^ Kobayashi et al.^63^, and Longrich et al.^7^ and 14 new characters were added to help resolve lambeosaurine relationships, for a total of 380 morphological characters (see SI). Two characters were excluded from analysis (87 is made redundant by two new characters; 193 was added by a previous analysis but the character description was not specified). Geographic distribution was included in addition to morphology, because biogeography shows strong phylogenetic signal, with closely related species inhabiting the same landmass^110^. Biogeography was coded as a series of binary characters rather than a single, multi-state character^7^ since this allows implied weighting to assign each biogeographic character its own weight, rather than to assume all dispersals are equally probable. Four taxa were added; *Minqaria bata*, the Daoui hadrosaurid, the Sidi Chennane hadrosaurid, and *Tlatolophus galorum*.

Analyses were run in TNT^111^, using a new technology search algorithm and default settings for implied weighting (K = 3), and a strict consensus was estimated; analysis was repeated in PAUP 4.0 b10 in implied weighting and the same K = 3 under a heuristic search until 100,000 trees were recovered; this produced an identical strict consensus. TNT returned 7 trees with a score of 120.23435. The resulting strict consensus (Fig. 10) has a CI of 0.3669, an RI of 0.8244, and a rescaled consistency index of 0.3025 (for full tree, see SI).

### Supplementary Information

Supplementary Information.

## Data Availability

All data generated or analyzed during this study are available in the paper or as part of the associated supplementary information.
