# Supplementary figures and images for: A new small duckbilled dinosaur (Hadrosauridae: Lambeosaurinae) from Morocco and dinosaur diversity in the late Maastrichtian of North Africa
Source: Sci Rep. 2024 Feb 13;14:3665. doi: 10.1038/s41598-024-53447-9 (PMC10864364; doi:10.1038/s41598-024-53447-9)

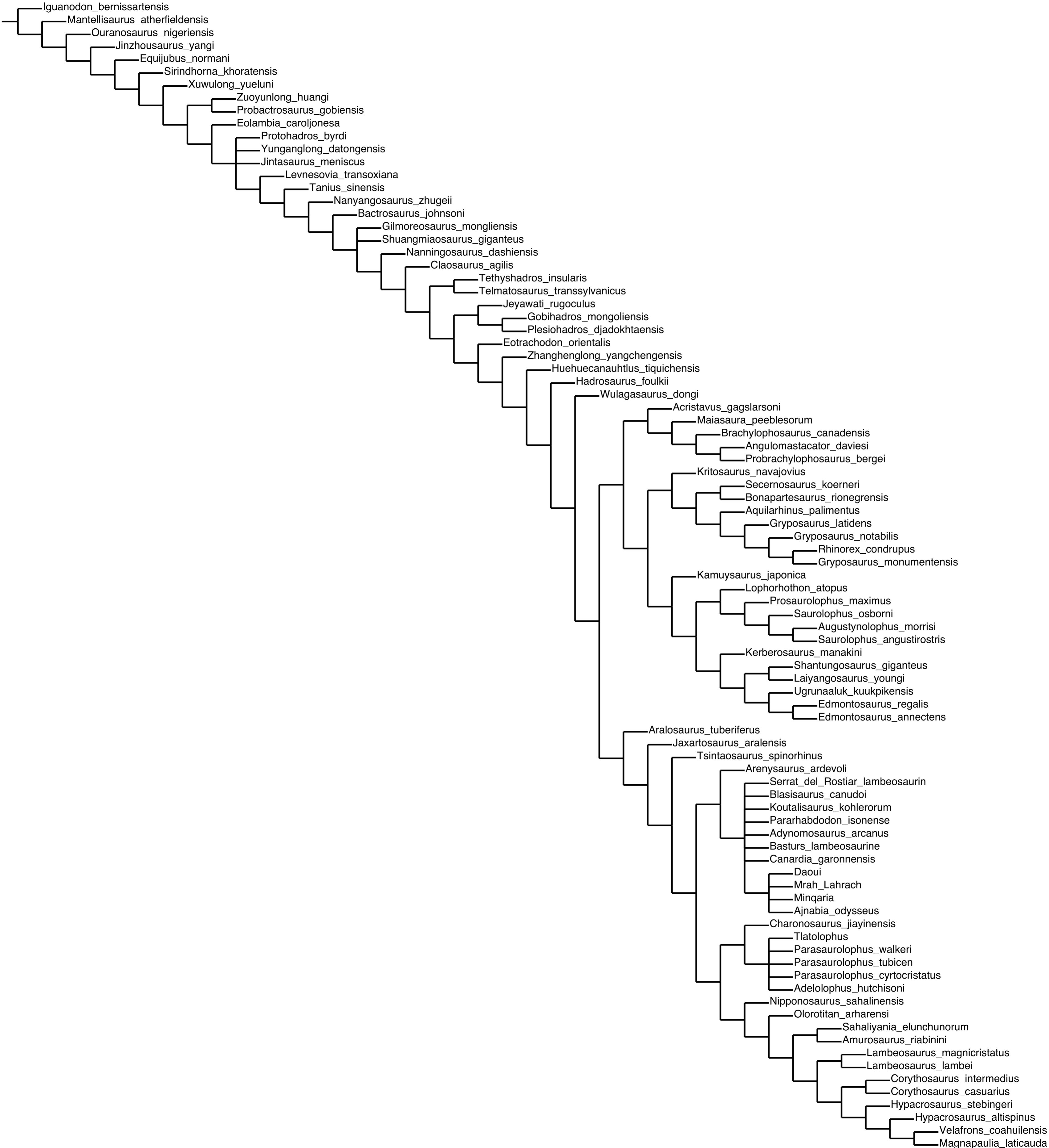

Supplement: Supplementary file 1 — Supplementary Information. [file 41598_2024_53447_MOESM1_ESM.zip › SI Figure 1 tree.pdf]
